# Alteration of stool pH and its association with biomarkers of gut enteropathy among slum-dwelling women of reproductive age in Bangladesh

**DOI:** 10.1186/s12905-023-02758-6

**Published:** 2023-12-09

**Authors:** Ishita Mostafa, S. M. Tafsir Hasan, Md. Amran Gazi, Md. Ashraful Alam, Shah Mohammad Fahim, Kazi Nazmus Saqeeb, Tahmeed Ahmed

**Affiliations:** 1 International Centre for Diarrhoeal Disease Research, Bangladesh (icddr,b), 68, Shaheed Tajuddin Ahmed Sarani, Mohakhali, Dhaka, 1212 Bangladesh; 2 Office of the Executive Director, icddr,b, Dhaka, 1212 Bangladesh; 3 Department of Global Health, University of Washington, Seattle, WA 98195 USA; 4 Department of Public Health Nutrition, James P Grant School of Public Health, BRAC University, Dhaka, 1212 Bangladesh

**Keywords:** Stool pH, Gut health, Gut enteropathy, Women of reproductive age, Intestinal alkaline phosphatase, lipocalin-2

## Abstract

**Background:**

Recent evidence suggests that measures of maternal gut enteropathy are associated with unfavorable fetal outcomes. It is, therefore, crucial to identify and treat the features of intestinal enteropathy among reproductive-age women living in areas where enteropathy is highly prevalent. However, there is a lack of non-invasive diagnostic tests to determine EED, making it difficult to identify the disease in field settings. In this study, we tested the potential of fecal pH as a biomarker of gut enteropathy and investigated its relationship with fecal biomarkers of intestinal enteropathy in reproductive-age women living in resource-limited environments.

**Methods:**

Data on socio-demographic information, anthropometry, and biological samples were collected from 78 apparently healthy women aged between 20 and 27 years from November 2018 to December 2019. The association of stool pH with two fecal biomarkers of gut enteropathy (i.e., intestinal alkaline phosphatase [IAP] and fecal lipocalin-2 [LCN-2] was investigated using multiple linear regression models after adjusting for relevant covariates.

**Results:**

In the adjusted models, alkaline stool pH (pH > 7.2) was found to be significantly associated with a decrease in the fecal IAP level by 1.05 unit (95% CI: -1.68, -0.42; p < 0.001) in the log scale, and acidic stool pH (pH < 6) was found to be significantly associated with an increase in the fecal LCN-2 level by 0.89 units (95% CI: 0.12, 1.67; p < 0.025) in the log scale.

**Conclusions:**

The study findings demonstrated an association of fecal pH with biomarkers of gut enteropathy indicating its applicability as a simple tool for understanding intestinal enteropathy among reproductive-age women living in resource-limited settings.

## Introduction

The growth and development of the offspring are interlinked with maternal health and nutritional status [1–5]. The World Health Assembly’s 2012 global nutrition target places a strong emphasis on interventions that aim to prevent undernutrition in the first 1000 days of life. Therefore, the global interest in maternal determinants of childhood undernutrition has recently increased. These determinants include maternal education, maternal age, and maternal health status. Addressing these factors can significantly improve the nutritional status of children in their early years of life [6]. Emerging data indicate that measures of maternal gut enteropathy are associated with unfavorable perinatal outcomes, including preterm birth, low birth weight (LBW), and small gestational age (SGA) at birth [7–9]. During pregnancy, maternal gut microbiota can be affected by factors such as maternal diet, obesity, stress and depression, infection, and medicines which are correlated with gender-specific differences in fetal development [10]. When maternal gut microbial homeostasis is disrupted, it leads to an imbalance of bacterial composition defined as dysbiosis. Dysbiosis can be due to the loss of beneficial bacteria, overgrowth of potentially pathogenic bacteria, and loss of overall bacterial diversity [11]. The dysbiosis in the maternal gut microbial community can alter offspring’s microbiota and immunity through vertical transmission which later contributes to the risk of non-communicable diseases in adulthood [12–15]. Thus, early detection of maternal gut inflammation might be helpful in early intervention resulting in better nutritional outcomes in the offspring.

Studies have shown a linkage between gut microbiota dysbiosis and intestinal inflammation caused by environmental enteric dysfunction (EED) [16, 17]. EED is a clinical condition marked by subclinical inflammation in the small intestine, blunting of the villi, and a decrease in the capacity of the intestines to absorb food. This condition is frequently found among individuals chronically exposed to enteropathogens due to residing in a contaminated environment with improper water, sanitation, and hygiene (WASH) conditions [18]. The Malnutrition and Enteric Disease (MAL-ED) study showed a high prevalence of EED among children in LMICs [19]. This condition in children can be carried into adulthood. In adults, EED usually presents only with sub-acute weight loss and is, therefore, hard to diagnose. The gold standard for the diagnosis of EED involves a biopsy of the intestinal tissue and confirmation by histopathology. The Bangladesh Environmental Enteric Dysfunction (BEED) study revealed that 95% of adults residing in slums, who are asymptomatic, exhibit chronic non-specific intestinal inflammation, a hallmark feature of EED [20]. However, collecting endoscopy-guided small intestinal biopsy samples from clinically asymptomatic adults for routine diagnosis of EED is technically infeasible and, in some instances, unethical [20–22].

Thus, interest has grown in non-invasive indirect biomarker evaluation of intestinal inflammation utilizing enzyme tests (ELISA). Recently, fecal biomarkers have been evaluated as a feasible noninvasive tool to assess intestinal inflammation [23–25], and studies have established the association of several fecal biomarkers with intestinal inflammation [26–29]. IAP and fecal LCN-2 are among the promising fecal biomarkers of interest [30, 31]. IAP is an endogenous protein and a member of the alkaline phosphatase family. It plays a vital role in regulating intestinal inflammation [32]. LCN-2, also known as neutrophil gelatinase-associated lipocalin, is a bacteriostatic peptide. It might be regarded as a broadly dynamic marker of intestinal inflammation, as according to a study, the levels of fecal LCN-2 were found to increase by more than ten times in response to Dextran Sodium Sulfate (DSS) levels that caused mild or low-grade inflammation, and by over ten thousand times in response to DSS levels where the presence of colitis is histopathologically apparent [33]. These biomarkers have the potential to be used in predicting or diagnosing EED [34].

A study examined the factors associated with undernutrition among slum-dwelling adults in Bangladesh. The results show that adults living in Bangladeshi slums are more likely to be malnourished and have a number of physiological and sociodemographic problems, including gastrointestinal inflammation and changes in intestinal permeability. Biomarkers of intestinal inflammation were also higher in both male and female adults [35]. However, these newer biomarker assays require a sophisticated lab and trained technicians, resulting in few investigations on intestinal inflammation in asymptomatic adults in LMICs. There is a constant quest to identify potentially inexpensive, noninvasive, simple-to-do, and sensitive assays to detect maternal gut inflammation and EED in its preclinical stage.

Studies have demonstrated that a simple technique like stool pH estimation can give us a good surrogate estimation of the overall gut environment [36, 37]. According to studies, variations in fecal pH can be linked to a variety of disease states, and extreme deviation from the normal range is associated with increased morbidity and mortality [38–41].

Therefore, measuring fecal pH can be a simple and cost-effective way to monitor gut health and potentially identify individuals at risk for certain diseases. However, further research is needed to determine the optimal range of fecal pH values for maintaining overall health and preventing disease. A low-cost, ready-to-use technique to identify many intestinal illnesses is stool pH. The relationship between stool pH and fecal indicators of gut inflammation in women of reproductive age has to be further investigated. In this study, slum-dwelling women were examined for their stool pH and two fecal indicators of gut inflammation, IAP and LCN-2.

## Methods

### Study site, participants, and data collected

The data for the current study were obtained from a clinical trial conducted from November 2018 to December 2019 in a slum in the Mirpur area of Dhaka city, Bangladesh. This place was selected as the study site because it is inhabited by poor and lower middle-income families, densely populated, and frequently lacks access to essential services like clean water, sanitation, electricity, and healthcare, which can be compared to any typical congested urban settlement. The trial titled “The microbiota-directed complementary food formulation (MDCF) primary MAM study (Clinical Trial Registration Number NCT04015999) was a community-based clinical trial where nutritional interventions (MDCF and ready to use supplementary food) were given to 124 moderately acute malnourished (MAM) children for a period of 3 months with an aim to improve their nutritional status and gut microbiota composition. The details of the study design and the results of the trial have been published elsewhere [42]. All 124 mothers of the children included in the primary MAM study were approached to participate in this study. However, only 80 of them consented to take part, and written informed consent was obtained from each of them. Out of the total sample size of 80 participants, 78 non-pregnant, non-lactating women of reproductive age were included in the data analysis. Two participants were excluded from the data analysis as later it was found out that they were pregnant at that particular time. As a part of the MDCF primary MAM trial, we obtained socio-demographic, WASH, hand hygiene practice-related information, and information on toilet facilities. Sanitation facilities include a pit latrine with a slab, a ventilated improved pit latrine, or a water-sealed septic tank to avoid underground infiltration considered as improved toilet facility. We have also performed anthropometric assessments, and collected fecal samples once at baseline from the women. The weight, height and BMI of the women were measured using a standard protocol [43].

### Biological sample collection and assay

Fecal samples were collected from the participants in sterile stool collection pots and were transferred to the laboratory, maintaining a cold chain. Stool pH was measured from the freshly collected stools. A portable stool pH meter (Hanna Instruments, Woonsocket, Rhode Island, USA) was used for the stool pH measurement. For measuring stool pH, 1 gm of stool was transferred to a separate sterile container, and a homogenized stool solution was prepared by adding ten milliliters of deionized water. The pH meter probe was then submerged in the solution and kept for a minute.

The remaining stool aliquots were kept at -80^o^C until further analysis. For measuring the fecal biomarkers, stool samples were weighed, and double-distilled water (ddH_2_O) was added at a specified ratio. A homogenized stool suspension was prepared by mixing 1 mg of stool with 50 µL of stool dilution buffer and then shaking the mixture vigorously. The suspension was then centrifuged for 20 min at a rate of 10,000Xg, and the IAP-containing supernatant was collected. Alkaline Phosphatase Diethanolamine Activity Kit was used to measure IAP activity following the manufacturer’s instructions (Sigma-Aldrich, St. Louis, USA). The stool IAP values are reported in U/ml. Fecal LCN-2 levels were detected using available ELISA kits (R&D system, Minneapolis, USA). The stool LCN-2 values are reported in ng/ml unit.

All laboratory analyses were conducted at the parasitology laboratory of icddr,b.

### Covariates

We have identified the covariates based on literature search and biological plausibility. A number of variables were considered including age, BMI, level of education, religion, monthly family income, the number of family members, and variables related to hand hygiene and wash practices [29, 35, 44, 45].

### Statistical analysis

We presented the characteristics of the women using mean and standard deviation for continuous variables and frequency measures for categorical variables. IAP activity and LCN-2 concentration was log-transformed for further analysis because they were log-normally distributed. Based on evidence from published articles, stool pH was categorized into three groups, acidic (pH < 6.0), normal range (6.0 ≤ pH ≤ 7.2), and alkaline (pH > 7.2) [46]. We visualized the distribution of log-transformed IAP activity and LCN-2 concentration in the women’s fecal samples using box plots across the ranges of stool pH. Separate simple and multiple linear regression models were built for each of the biomarkers (log-transformed IAP and LCN-2) to assess the association of each fecal biomarker with stool pH. Strength of association was expressed as β (mean difference) with 95% confidence interval (CI). In the multivariable models, all the covariates of a priori interest were included. Statistical significance was set at p < 0.05. All the analyses were performed using STATA V.13.

## Results

Women’s characteristics are reported in Table 1.

**Table 1:** Baseline characteristics of the study participants (n = 78)

Indicators	Number (percentage)
Age (in years), Mean (SD)	24.1 (4.9)
Weight (in kg), Mean (SD)	48.1 (9.4)
Height (in cm), Mean (SD)	149.6 (5.1)
**BMI Category**	
< 18.5 kg/m^2^	19 (24.4)
≥ 18.5 to < 25 kg/m^2^	46 (59.0)
≥ 25 kg/m^2^	13 (16.7)
**Religion**	
Islam	75 (96.2)
Hinduism	3 (3.9)
**Educational status**	
Primary incomplete	20 (25.6)
Primary complete	18 (23.1)
Secondary incomplete	20 (25.6)
Secondary complete or higher	7 (9.0)
Only can write her name	13 (16.7)
No. of family members, Mean (SD)	5.1 (2.6)
Monthly family income in USD*, Mean (SD)	163.7 (94.0)
Use soap for hand wash after defecation	11 (14.1)
Treatment of drinking water (yes)	29 (37.2)
Toilet facility (improved)	56 (71.8)
**Stool pH level**	
pH < 6	28 (35.9)
pH ≥ 6 and ≤ 7.2	33 (42.3)
pH > 7.2	17 (21.8)
IAP activity (U/ml) in log scale, Mean (SD)	5.2 (0.96)
LCN2 (ng/ml) in log scale, Mean (SD)	5.5 (1.5)

*1 USD = 106 BDT

The mean age of the women was 24.1 years, ranging from 20 to 27 years. A large majority of the women were Muslim (96.2%). The mean family income was 163.7 USD per month. More than a quarter of the participants had secondary-level education (25.6%). Most of the participants do not treat water before drinking (62.8%). Among the women, 14.1% used soap for hand washing after defecation. More than half of the participants used improved toilet facilities. The women’s stool pH was abnormal (35.9% had acidic pH and 21.8% had alkaline pH).

**Fig. 1:**
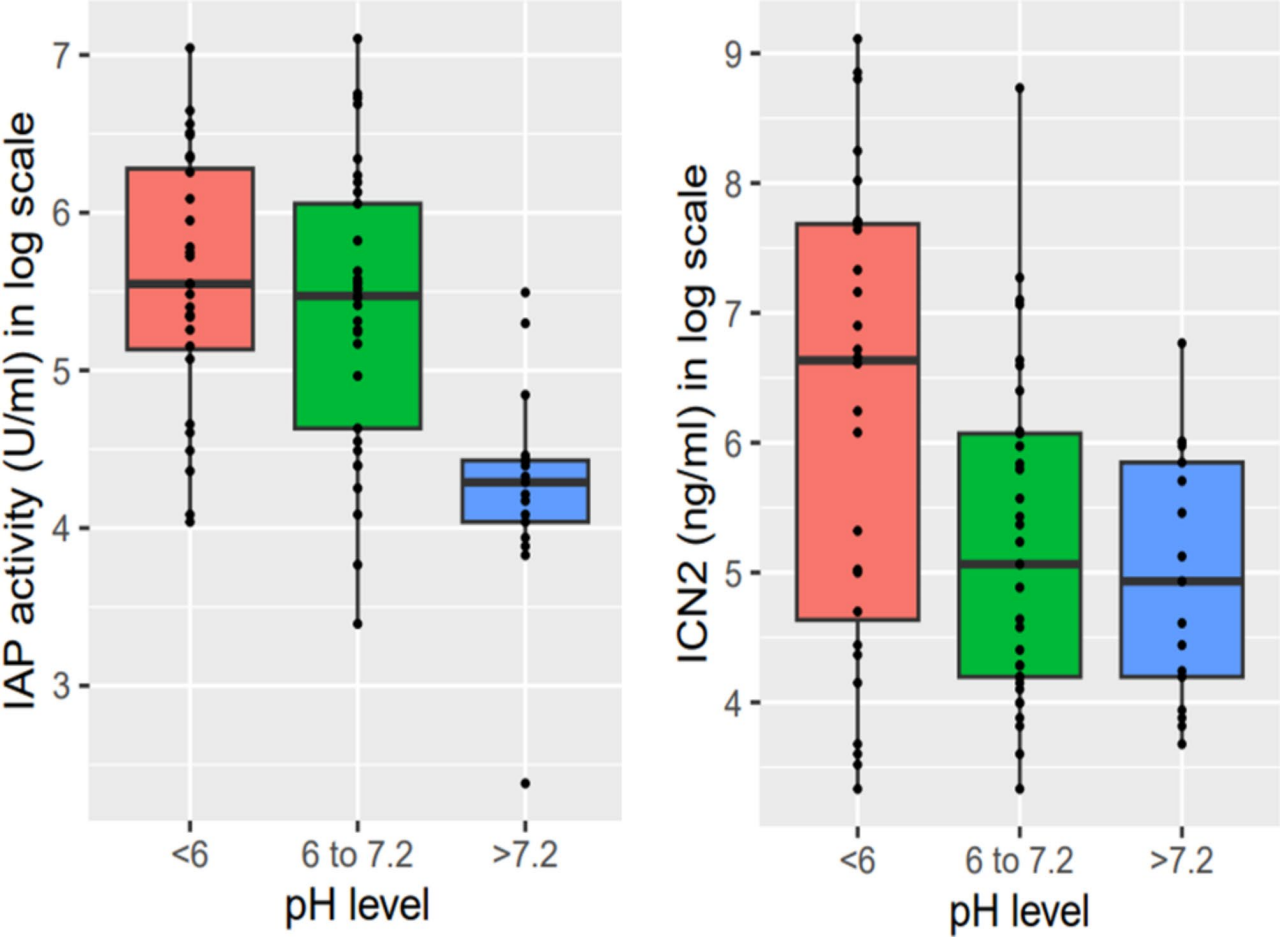
Box plot representation of fecal biomarkers with change in stool pH level

The level of the fecal biomarkers seemed to vary with the change in stool pH level (Fig. 1).

In the adjusted models, alkaline stool pH (pH > 7.2) was found to be significantly associated with decrease in the fecal IAP level by 1.05 unit (95% CI: -1.68, -0.42; p < 0.001) in the log scale (Table 2) and.

**Table 2:** Association of fecal pH level with Log IAP

Variable	Unadjusted		Adjusted*	
	β (95% CI)	p-value	β (95% CI)	p-value
pH < 6	0.18 (-0.24, 0.61)	0.386	0.21 (-0.28, 0.70)	0.394
pH > 7.2	-1.15 (-1.64, -0.66)	< 0.001	-1.05 (-1.68, -0.42)	0.002
pH ≥ 6 to ≤ 7.2 was the reference category				

*Adjusted for Age, BMI category, Education category, Monthly family income (in USD), Number of family members, Type of toilet used, Use of soap after defecation, Treatment of drinking water category

acidic stool pH (pH < 6) was found to be significantly associated with increase in fecal LCN-2 level by 0.89 units (95% CI: 0.12, 1.67; p < 0.025) in the log scale. (Table 3).

**Table 3:** Association of fecal pH level with Log LCN2

Variable	Unadjusted		Adjusted*	
	β (95% CI)	p-value	β (95% CI)	p-value
pH < 6	1.01 (0.27, 1.74)	0.008	0.89 (0.12, 1.67)	0.025
pH > 7.2	-0.25 (-1.10, 0.60)	0.558	-0.16 (-1.16, 0.83)	0.745
pH ≥ 6 to ≤ 7.2 was the reference category				

*Adjusted for age, BMI category, education category, monthly family income (in USD), number of family members, type of toilet used, use of soap after defecation, treatment of drinking water category

## Discussion

The findings of our investigation indicate a significant association between alterations in fecal pH levels and biomarkers of gut enteropathy, specifically LCN-2 and IAP, demonstrating either a drop or rise from neutral level of stool pH, respectively. While previous research demonstrated a notable correlation between fecal pH and severe acute malnutrition [47], a comprehensive examination of the existing literature did not provide any findings regarding the connection between fecal pH and indicators of EED in humans.

The results obtained from this study could have significant implications for developing countries where access to expensive diagnostic tools is limited. As sample collection and preparation is very simple and stable, in settings with limited resources, stool pH testing may provide a cost-effective way and can be used as a potentially affordable and simple first-line investigation to identify intestinal inflammation, leading to earlier treatment and improved outcomes. The expenditure associated with conducting ELISA using commercially available kits for the quantification of EED biomarkers is around two to three times more in comparison to using a portable pH meter for measuring the stool pH. Furthermore, the feasibility of conducting this stool pH testing directly at the site of stool collection is an advantage, as it may be performed by individuals with minimal training. In contrast, the ELISA method necessitates the involvement of highly skilled staff.

Our current study revealed that highly acidic stool pH (< 6) was significantly associated with increased fecal LCN-2 level after adjusting for other covariates. LCN-2 plays a role in the body’s host immune system by limiting the growth of pathogenic bacteria in the gut [48]. Studies have shown that LCN-2 expression is increased in patients with intestinal inflammatory disorders, and its levels were significantly associated with the degree of severity of gut inflammation [49–53]. On the other hand, results from published studies also showed that inflammation in the gut villus caused by an increase in pathogenic gut bacteria resulted in a decrease in stool pH by interfering with the carbohydrate absorption pathway [54–57]. As there is a scarcity of research linking fecal LCN-2 level with the stool pH directly, it is therefore, difficult for us to compare our findings directly with other study findings. However, based on the above-mentioned findings, it can be said that the association of elevated levels of fecal LCN-2 with highly acidic stool pH are, in fact, in line with the published scientific works. Moreover, as fecal LCN-2 can be a promising biomarker to predict EED [34], stool pH might also be a low-cost tool to detect intestinal inflammation (EED) in asymptomatic adult individuals.

The findings of our study further showed that fecal levels of IAP were significantly negatively associated with highly alkaline stool pH (> 7.2) after adjusting for other covariates. In inflammatory conditions of the gut, expression of IAP is found to be decreased, and fecal IAP concentration is found to be lower in individuals suffering from chronic enteropathy. The relationship between IAP deficiency and overproduction and their effect on tight-junction protein (TJP) levels and function were studied. Results from studies suggested that IAP is a chief regulator of gut mucosal permeability and may act by improving TJP levels and localization [58]. IAP is a potential biomarker to monitor colitis in a mouse model of inflammatory bowel diseases (IBD). It was also reported that gut inflammation improved after treatment with synthetic IAP [59–63]. Even though there is a scarcity of human IAP-related studies, animal studies have shown that intestinal and fecal pH was higher among the IAP-knockout mice compared to their healthy counterparts [64], which is similar to our study findings.

Intestinal inflammatory disorders, which were previously believed to be the disease of the western world, are gradually increasing among the people of the developing world [65]. Many of the asymptomatic adults (both male and female) living in the unhygienic conditions of LMICs were suffering from intestinal inflammation. It is evident from published research that chronic low-grade inflammation in the gut can be a precursor for many health conditions, including adverse pregnancy outcomes in females [66, 67]. Early diagnosis and initiation of treatment are, therefore, pivotal for a better outcome of the disease. However, assessing gut inflammation using current techniques (Endoscopy, fecal biomarkers assessment, etc.) requires highly technical skills and a costly setup that is inappropriate for low-resource settings. Therefore, using stool pH as a proxy for fecal biomarkers (LCN-2, IAP) of intestinal inflammation would be highly beneficial for the early detection of enteric inflammation and assessing overall gut health in a resource-poor setting. In that context, we anticipate that the outcomes of our research will have a significant public health impact, especially for LMICs.

The findings of this study should be interpreted in light of its limitations since they are anticipated to have high public health importance for the early detection of enteric inflammation in women living in LMICs using a low-cost technique. The first drawback is the small sample size, and the second is that the study participants were not subjected to the gold standard confirmatory test for identifying intestinal inflammation (intestinal biopsy and histopathology) because of moral concerns. Besides, information on dietary intake and other co-morbidities were not collected from the study participants. Future studies with larger sample sizes and confirmatory tests could further validate these findings.

## Conclusion

The study findings demonstrated an association of fecal pH with biomarkers of gut enteropathy among reproductive-age women. This result suggests that fecal pH could serve as a potential measure for assessing enteropathy in individuals residing in resource-poor settings. Considering the low-cost and non-invasiveness of the test, stool pH can be assessed in any settings where enteropathy is highly prevalent. However, although the findings are promising, sufficiently powered and well-designed future studies are necessary to confirm the association of stool pH with fecal biomarkers of intestinal health and gut enteropathy (LCN-2, IAP), particularly among women of reproductive age.

## Data Availability

The datasets used and/or analyzed during the current study available from the corresponding author on reasonable request.
